# Smartphone Thermal Imaging for Preoperative Perforator Mapping in Perforator Based Flaps

**DOI:** 10.7759/cureus.51755

**Published:** 2024-01-06

**Authors:** Sarosh Ismail, Bushra Zulfiqar, Waqas Sami, Sadaf Gulzar, Faisal Akhlaq, Erum Naz, Sukaina Rupani

**Affiliations:** 1 Department of Plastic Surgery, Dow University of Health Sciences, Civil Hospital Karachi, Karachi, PAK; 2 Department of General Surgery, Dow University of Health Sciences, Civil Hospital Karachi, Karachi, PAK

**Keywords:** thermal camera, visual assessment, smartphone-based thermal imaging, perforators, flap

## Abstract

Objective: To determine the diagnostic accuracy of smartphone thermal imaging for preoperative perforator mapping in perforator-based flaps, taking visual inspection as gold standard.

Methodology: It was a cross-validation study conducted at the Department of Plastic Surgery, Dr. Ruth K. Pfau Civil Hospital, Karachi, Pakistan, from August 2022 to January 2023. All adult patients aged 18 to 40 years of either gender undergoing perforator flap surgery were included. Each patient followed the same treatment regimen, which involved the preoperative identification of the perforator location using the FLIR One camera. Subsequently, confirmation was achieved during the surgical procedure through visual inspection. A two-by two table was used to calculate sensitivity, specificity, positive predictive value, negative predictive value, and diagnostic accuracy.

Results: The mean age of the patients was 30.10±6.87 years, ranging from 18 to 40 years. Most of the patients were males (58.7%), and 41.3% were females. Almost 80.4% were pedicle flaps, and 19.6% were free flaps. The accuracy of thermal imaging was found to be 83.2%, with a sensitivity of 84.3%, a specificity of 80%, a PPV of 92.9%, and a NPV of 62.2%, respectively.

Conclusion: Smartphone-based thermal imaging is useful for the diagnosis of perforators and has high sensitivity and specificity.

## Introduction

Perforator-based flaps are one of the most effective reconstruction strategies, resulting in the development of many new flaps, each associated with new pedicles distributed throughout the body and all offering significant benefits. Perforator flaps are cutaneous and subcutaneous tissue patches that are supplied by perforator arterial branches that originate from the main vascular bundles and travel intramuscularly or intraseptally [1,2]. The efficacy of perforator-based flaps has been proven internationally [3,4]. However, the procedure requires a combination of factors that include surgical expertise, detailed knowledge of the anatomy, and exact perforator localization. Various studies have enabled a better understanding of vascular anatomy and skin circulation patterns. Preoperative perforator assessment offers the reconstructive surgeon a roadmap to help guide decision-making during flap raising, and it has surely contributed to the steady development of surgical techniques. Doppler ultrasonography, hand-held doppler ultrasound, computed tomography angiography, and, to a lesser extent, magnetic resonance angiography are currently the core objective assessment modalities for preoperative perforator planning [5-7].

Though all these techniques have made perforator flaps easier, they have also shown good outcomes in terms of complications and survival rate. However, limitations like highly advanced surgical skills, lack of accessibility due to non-portable equipment, administration of intravenous contrast, and financial constraints within low-resource healthcare settings, compounded by the need for highly specialized and trained personnel, have made these modalities difficult to use frequently [8,9].

Perforator flaps have become easier since the emergence of smartphone thermal imaging in this regard. Smartphone-based thermal imaging technology exploits the ability of perforator vessels to generate a detectable heat signal, discernible through infrared thermography, facilitating their localization [10,11]. Evidence suggests that smartphone thermal imaging offers a cost-effective, easily accessible, and non-invasive technique for pre-operative planning as well as intraoperative and post-operative assessment of flap perfusion [12,13]. However, smartphone-based thermal imaging is a new technique, so limited data is available on this topic from Pakistan. Therefore, the aim of the current study was to determine the diagnostic accuracy of smartphone thermal imaging for preoperative perforator mapping in perforator-based flaps, taking visual inspection as the gold standard.

## Materials and methods

It was a cross-validation study conducted at the Department of Plastic Surgery, Dr. Ruth K. Pfau Civil Hospital, Karachi, Pakistan, from August 2022 to January 2023. The sample size was calculated using an online sample size calculator by Wan Nor Arifin [14]. The sample size of 143 was calculated using sensitivity as 86.2%, specificity as 80%, prevalence as 57%, absolute precision as 10%, and confidence level as 95% [15]. All adult patients aged 18 to 40 years of either gender undergoing perforator flap surgery were included. All patients with any conditions-such as acute inflammation, osteomyelitis, vasculitis, chronic kidney or liver illness, gross heart dysfunction, or usage of vasoactive drugs (nitroglycerin, beta blockers, and calcium channel blockers)-that could change tissue temperatures or patients with hyperthermia (>99.5oF) or hypothermia (<98.6oF) were excluded.

The study was conducted after getting approval from the institutional review board of Dow University of Health Sciences (Ref# IRB-2303/DUHS/Approval/2022/942). Signed informed consent was obtained from all study participants after explaining the pros and cons of the study.

The day before surgery, a pre-operative examination, thermal camera, and hand-held doppler-aided marking and photography were performed. The flap was raised intraoperatively, and perforators were found. The dominant perforators were pulsatile or had a bigger size than the smaller ones. Photographs taken during and after surgery, as well as discoveries, were kept on file.

The extremity and wound were exposed, and the room temperature was maintained at 22°C by keeping the thermostat of the heat ventilation and conditioning system at this temperature. The primary investigator employed a thermal imaging camera based on a mobile phone, the FLIR ONE®. The timer was started, and the first image was taken after one minute, which was the amount of time required to bring the surface temperature into balance with the ambient temperature. The skin surface was dried, and the image was captured. Then, until the thermal picture showed a uniformly cold surface, an ice pack was applied. Once hot areas formed after the ice pack was removed, a thermal image was taken to mark the locations of prominent perforators. The countdown was stopped once the perforator areas had been indicated. Brighter areas that arrived before the smaller, less luminous dots were regarded as dominant. Photographs were taken, and any markings were wiped away. As a gold standard for evaluating the diagnostic accuracy of the thermal imaging camera, perforators were confirmed by per-operative ocular or visual inspection. Data regarding age, gender, co-morbidities, and perforator flap type were collected.

IBM Corp. Released 2016. IBM SPSS Statistics for Windows, Version 24.0. Armonk, NY: IBM Corp. was used for the purpose of statistical analysis. The mean and standard deviation were computed for quantitative variables like the age of the patients. Frequencies and percentages were calculated for qualitative variables like gender, comorbidities, type of perforator-based flap, thermal scan findings, and visual inspection findings. True positives were perforators on thermal imaging that matched visual inspection findings. False positives were perforators found on thermal imaging but not on visual inspection. False negatives were perforators found on visual inspection but not on thermal images. True negatives were perforators neither found on thermal images nor on visual inspection. A two-by two table was used to calculate sensitivity, specificity, positive predictive value, negative predictive value, and diagnostic accuracy.

## Results

The mean age of the patients was 30.10±6.87 years, ranging from 18 to 40 years. Most of the patients were males (n=84, 58.7%), and 59 (41.3%) were females. Of 143 patients, 10 (7%) had hypertension, six (4.2%) had diabetes, and three (2.1%) had paraplegia, respectively. Almost 115 (80.4%) were pedicled flaps, and 28 (19.6%) were free flaps (Table 1).

**Table 1:** Baseline characteristics of study participants (n=143) The data is presented as n (%) or Mean±SD.

Characteristics	Statistics
Age (years)	30.10±6.87
Gender	
Male	84 (58.7)
Female	59 (41.3)
Comorbids	
Hypertension	10 (7)
Diabetes	6 (4.2)
Paralagia	3 (2.1)
Region of flap	
Upper limb	22 (15.3)
Lower limb	31 (21.7)
Abdomen	55 (38.5)
Groin	35 (24.5)
Type of flap	
Free flap	28 (19.6)
Pedicled flap	115 (80.4)

Perforator flaps included in this study were the deep inferior epigastric artery perforator flap, the abdominal perforator flap, the thoracodorsal artery perforator flap, the anterior interosseous artery perforator flap, the posterior tibial artery perforator flap, and the superficial circumflex iliac artery perforator flap.

Out of 143, 98 (68.5%) perforators were detected on thermal imaging, and 108 (75.5%) were detected on visual inspection. The accuracy of thermal imaging was found to be 83.2%, with a sensitivity of 84.3%, a specificity of 80%, a PPV of 92.9%, and a NPV of 62.2% (Table 2).

**Table 2:** Diagnostic accuracy of thermal imaging for detecting perforators by taking visual inspection as the gold standard (n=143) The data is presented as n (%).

Thermal imaging	Visual inspection		Total	
	Yes	No		
Yes	91 (84.3%)	7 (20%)	98 (68.5%)	
No	17 (15.7%)	28 80%)	45 (31.5%)	
Total	108 (100%)	35 (100%)	143 (100%)	
Sensitivity=84.3%, Specificity=80%, PPV=92.9%, NPV=62.2%, Accuracy=83.2%				

## Discussion

The use of smartphone-based thermal imaging for preoperative perforator mapping in perforator-based flaps is a relatively new and promising approach, aiming to overcome the limitations associated with traditional techniques such as Doppler ultrasonography, computed tomography angiography, and magnetic resonance angiography [16,17]. These traditional techniques require highly specialized skills and experience, access to non-portable equipment, the administration of intravenous contrast agents, and often significant financial resources. Moreover, in resource-constrained healthcare settings, these limitations can further restrict the use of these techniques [18,19]. Whereas smartphone thermal imaging leverages the heat generated by perforator vessels, making it detectable through infrared thermography. This technology has the potential to provide a cost-effective, easily accessible, and non-invasive means of preoperative planning, intraoperative guidance, and post-operative assessment of flap perfusion [17]. The limitation of using this device is the use of an ice pack and patient discomfort related to it. Further comparison and studies using an ice pack and some other skin cooling agent like alcoholic disinfection, a wet laparotomy sponge, or simple fan cooling that is less uncomfortable for the patient might be helpful in the future.

In the current study, the diagnostic accuracy of thermal imaging for the detection of perforators was 83.2%, the sensitivity was 84.3%, and the specificity was 80%. Similarly, in another Pakistani study by Rabbani et al., the sensitivity of thermal imaging was 86.2%, and the specificity was 80% [15]. In the study by Muntean et al., the diagnostic accuracy of thermal imaging was 77.4%, with sensitivity of 95% and positive predictive value of 81%, respectively [20]. Weum et al. concluded that perforators are adequately detected on thermal imaging and have a relatively high accuracy of 96% [21]. In the study by Pereira et al., smartphone-based thermal imaging had 100% sensitivity and 98% specificity for the detection of cutaneous perforators by taking CT angiographs as a reference standard [22]. Sheena et al. also revealed that thermal imaging is an effective tool for detecting cutaneous lesions, with a 97% confirmation by hand-held Doppler [23]. Paul et al. found that the thermal imaging camera was able to capture accurately the thermal readings of photographic images, allowing for the identification of reliable perforator locations in the lower limbs [24]. Hardwicke et al. also confirmed that FLIR ONE smartphone-based thermal imaging is a cost-effective and reliable technique in the field of plastic surgery [25].

**Figure 1:**
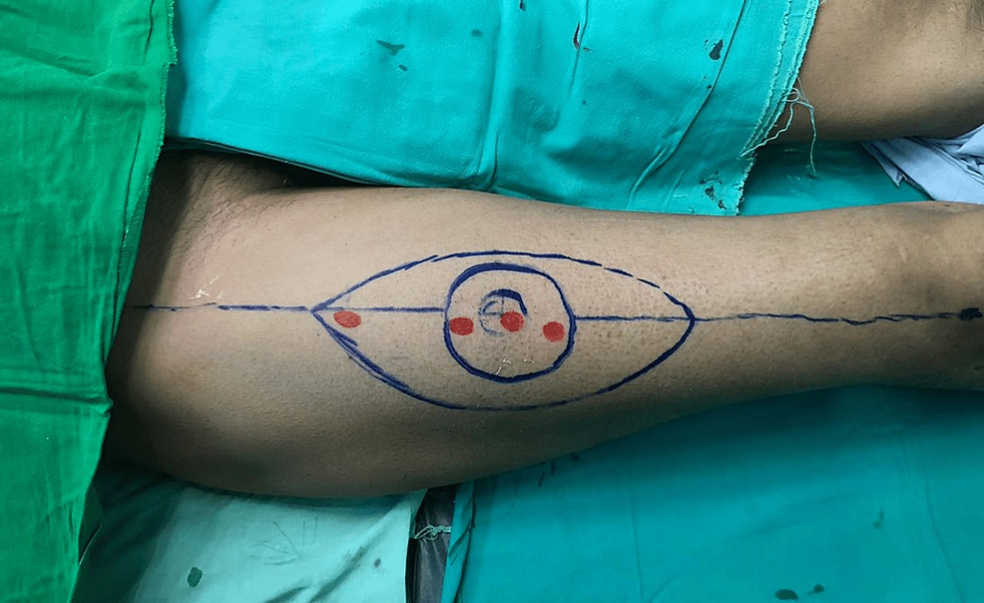
This marking is of ALTF for the patient undergoing reconstitution of a cheek defect secondary to buccal mucosa OSCC tumor excison. Marking was done one day before surgery; perforators from a hand-held doppler ultrasound were also marked. ALTF: Anterio-lateral thigh flap, OSCC: Oral squamous cell carcinoma

**Figure 2:**
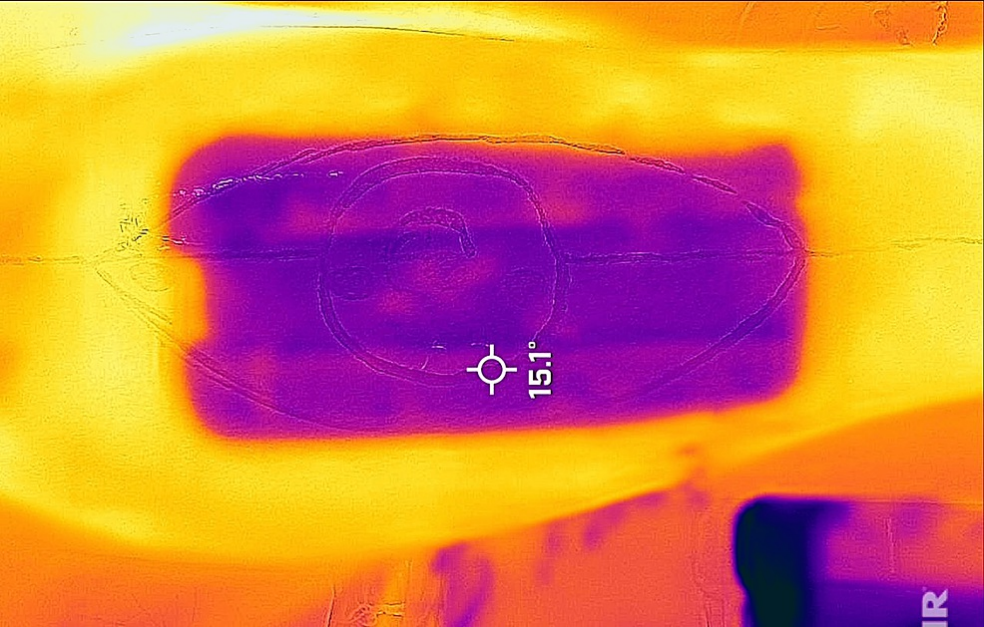
The ice pack was applied to the marked flap region after maintaining the temperature of the room at 22°C. This is the first image taken after removing the ice pack.

**Figure 3:**
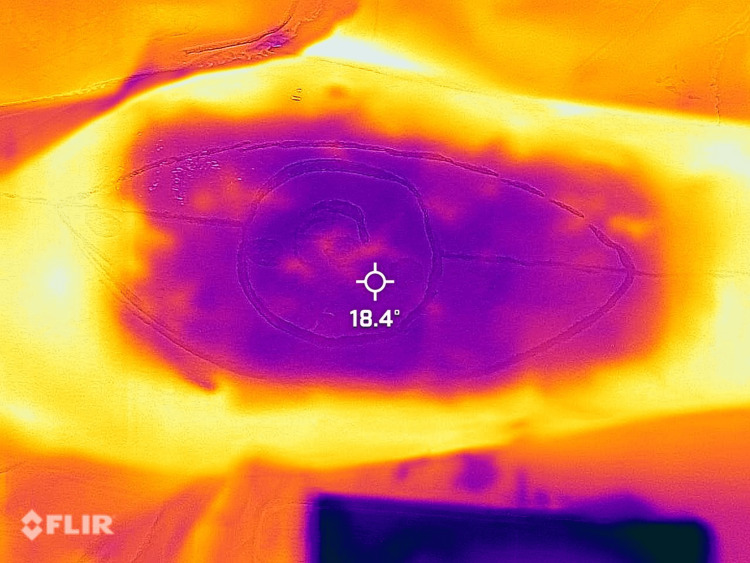
Bright yellow-colored spots appeared in the marked territory of flaps—brighter areas that arrived before the smaller, less luminous dots will be regarded as dominant.

**Figure 4:**
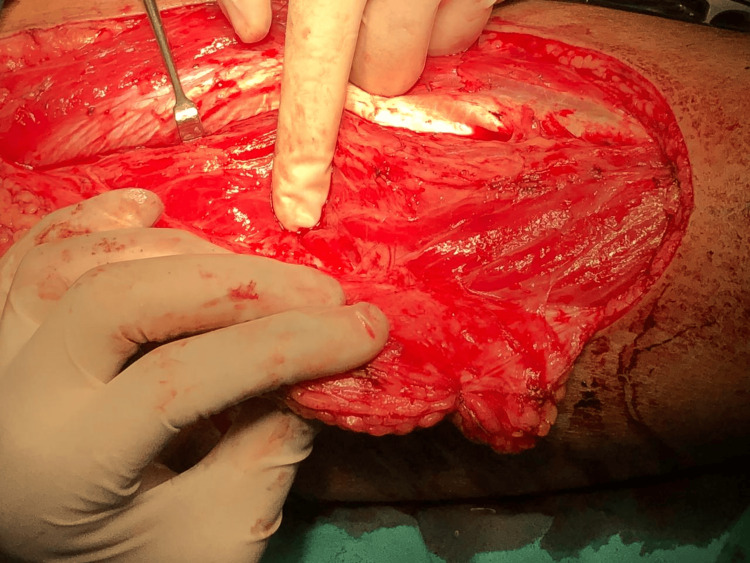
Perforators were confirmed by a preoperative visual inspection.

These findings suggest that smartphone thermal imaging is a valuable tool for preoperative perforator mapping, with a high sensitivity and specificity ensuring the reliable identification of perforators and a high accuracy indicating that positive findings on thermal imaging are likely to be accurate (Figures 1-4).

## Conclusions

With its high sensitivity and specificity, smartphone-based thermal imaging provides a practical, readily available, portable, and affordable method of diagnosing perforators. This noninvasive device helps to obtain the best possible outcome by giving the surgeon real-time imaging of the most appropriate perforator and enabling continuous monitoring of the flap perfusion at various stages before and following flap harvest.
